# Potentiometric determination of moxifloxacin in some pharmaceutical formulation using PVC membrane sensors

**DOI:** 10.1186/s13065-014-0059-y

**Published:** 2014-09-17

**Authors:** Mohammed M Hefnawy, Atef M Homoda, Mohammed A Abounassif, Amer M Alanazi, Abdulrahaman Al-Majed, Gamal A Mostafa

**Affiliations:** Pharmaceutical Chemistry Department, College of Pharmacy, King Saud University, P.O.Box 2457, Riyadh, 11451 Saudi Arabia; Micro-analytical Lab, National Research Center, Dokki, Cairo, Egypt

**Keywords:** Moxifloxacin HCl, Sodium tetraphenyl borate, Phosphomolybdic acid, Phosphotungstic acid, PVC, Potentiometry

## Abstract

**Background:**

The construction and electrochemical response characteristics of Poly (vinyl chloride) membrane sensors for moxifloxacin HCl (MOX) are described. The sensing membranes incorporate ion association complexes of moxifloxacin cation and sodium tetraphenyl borate (NaTPB) (sensor 1), phosphomolybdic acid (PMA) (sensor 2) or phosphotungstic acid (PTA) (sensor 3) as electroactive materials.

**Results:**

The sensors display a fast, stable and near-Nernstian response over a relative wide moxifloxacin concentration range (1 × 10^−2^ - 4.0 × 10^−6^, 1 × 10^−2^ - 5.0 × 10^−6^, 1 × 10^−2^ - 5.0 × 10^−6^ M), with detection limits of 3 × 10^−6^, 4 × 10^−6^ and 4.0 × 10^−6^ M for sensor 1, 2 and 3, respectively over a pH range of 6.0 - 9.0. The sensors show good discrimination of moxifloxacin from several inorganic and organic compounds. The direct determination of 400 μg/ml of moxifloxacin show an average recovery of 98.5, 99.1 and 98.6% and a mean relative standard deviation of 1.8, 1.6 and 1.8% for sensors 1, 2 and 3 respectively.

**Conclusions:**

The proposed sensors have been applied for direct determination of moxifloxacin in some pharmaceutical preparations. The results obtained by determination of moxifloxacin in tablets using the proposed sensors are comparable favorably with those obtained using the US Pharmacopeia method. The sensors have been used as indicator electrodes for potentiometric titration of moxifloxacin.

## Background

**Figure 1:**
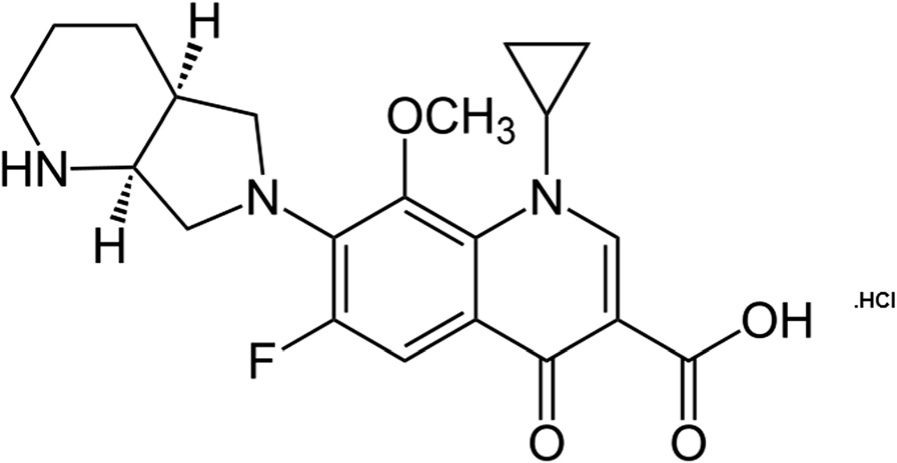
**Chemical structure of moxifloxacin hydrochloride.**

Moxifloxacin (MOX) (1-cyclopropyl-6-fluoro-1,4- dihydro-8-methoxy-7-[(4*aS*,7*aS*)-octahydro-6H-pyrrolo [3,4-b]pyridin-6-yl]4-oxo-3 quinoline carboxylic acid) (Figure 1) is an advanced-generation, 8-methoxyquinolone derivate of fluoroquinolone antibacterial agent that is synthetic. It was discovered in 1999 [1,2]. Moxifloxacin is a broad-spectrum antibiotic that is active against both Gram-positive and Gram-negative bacteria. It functions by inhibiting DNA gyrase, a type II topoisomerases, and topoisomerase IV [3], enzymes necessary to separate bacterial DNA, thereby inhibiting cell replication.

Various methods cited in literature for its determinations involve, spectrophotometry [4,5], spectrofluorimetry [6], atomic absorption spectrometry [7], conductometry [7], voltammetry [8], high performance liquid chromatography- ultraviolet (HPLC-UV) [9-11], HPLC-fluorescence (HPLC-Fl) [12-14] capillary electrophoresis (CE) [15,16], and HPLC-mass spectrometry (HPLC-MS) [17,18]. However, most of these methods involve time-consuming procedures, derivatization and/ or sophisticated instruments.

Due to the fact that MOX is a compound of great pharmacological and analytical importance, in recent years, there has been an increased interest to develop accurate analytical methods which are valid for quantification of MOX in biological and pharmaceutical samples.

Potentiometric methods, using ion selective electrodes, have found wide application [19-21] being simple, economical, applicable over a wide range of different areas, with applicability to turbid and colored solutions, and offering enough selectivity towards the drug in the presence of various pharmaceutical excipients. To the best of our knowledge till now no potentiometric membrane sensors for MOX have been published. The proposed sensors are based on the use of PVC membrane sensor of MOX - tetraphenylborate or MOX-phosphomolybdate or MOX-phosphotungstate as electroactive materials. The present work describes the construction and evaluation of novel PVC electrochemical sensors for the sensitive and selective determination of moxifloxacin in its pharmaceutical preparations.

### Experimental

#### Apparatus

All potentiometric measurements were made at 25 ± 1°C unless otherwise stated using an Orion pH/mV meter (model 330) using MOX membrane sensors in conjunction with an Orion double junction Ag/AgCl reference electrode (model 90–02) containing 10% (w/v) potassium nitrate in the outer compartment. Adjustment of pH was made with a combined Ross glass pH electrode (Orion 81–02) for all pH measurements.

#### Reagents and materials

All chemicals used were of analytical reagent grade unless otherwise stated and doubly distilled water was used throughout. Polyvinyl chloride powder (PVC) high molecular weight, dibutyl sebacate (DBS), dioctyl phthalate (DOP), o-nitrophenyl octylether (NPOE), tetrahydrofurane (THF) of purity > 99% were obtained from Aldrich Chemical Company and MOX was obtained from Sigma Chemical Company, Germany. Sodium tetraphenylborate (NaTPB), phosphomolybdic acid (PMA) and phosphotungstic acid (PTA), were obtained from BDH, Chemical Ltd. Avelox 400 mg, Manufactured by Bayer was obtained from local pharmacy. The stock solution of 1 × 10^−2^ M MOX was prepared by dissolving the appropriate amount of MOX in 100 ml of water. Five standard MOX solutions were prepared in the range of 1 × 10^−2^ - 1 × 10^−6^ M by diluting the appreciate amount in double distilled water. Tris buffer of pH 7.0 was prepared by mixing 100 ml of 0.1 M tris(hydroxymethyl) aminomethane hydrochloride, with appropriate 0.1 M HCl.

#### Preparation of the MOX-PVC membrane sensors

Upon the addition of 25 ml of 1 × 10^−2^ M of MOX solution to 25 ml each of 1 × 10^−2^ M sodium tetraphenyl borate or 75 ml of 1 × 10^−2^ M of MOX solution to 25 ml phosphotungstic acid respectively, a whitish precipitate of MOX-TPB or yellowish precipitate of MOX-PA or MOX-PT were formed, respectively. The precipitate was filtered off through a Whatman filter paper No. 42, washed with cold deionized water until no chloride ion was detected into the washing solution. The precipitate was dried under vacuum for 48 h, then grinded to a fine powder in mortar, forming ion-pairs complex. The elemental analysis confirmed the formation of 1:1 or 3:1 complex of MOX:TPB or MOX:PM or MOX:PT, respectively. Portion of ten mg of the prepared ion associate complexes were thoroughly mixed with 190 mg PVC powder, 350 mg of DBS or DOP or NPOE and 5 ml THF in glass Petri dishes (5 cm diameter). After the constituents being well mixed, the solvent has been allowed to evaporate overnight while the sensing membranes have been formed. The PVC master membranes were sectioned with a cork borer (10 mm diameter) and glued to a polyethylene tube (3 cm length, 8 mm I.D.) using THF [22,23]. Laboratory made electrode bodies were used, which consisted of a glass tube, to which the polyethylene tube is attached at one end and filled with internal reference solution (equal volumes of 1 × 10^−2^ M aqueous solution of MOX and KCl). Ag/AgCl internal reference electrode (1.0 mm diameters) was used. The indicator electrode was conditioned by soaking in a 1 × 10^−2^ M aqueous MOX solution for 1 h and stored in the same solution when not in use.

#### Procedure

The moxifloxacin PVC membrane sensors were calibrated by immersion in conjunction with the reference electrode in a 50 ml beaker containing 9.0 ml of tris-buffer of pH 7.0. Then 1.0 ml aliquot of MOX solution was added with continuous stirring, to give final MOX concentration ranging from 1 × 10^−2^ to 1 × 10^−6^ M and the potential was recorded after stabilization to ± 0.5 mV. A calibration curves were constructed by plotting the recorded potentials as a function of -log [MOX]. The resulting graphs were used for subsequent determination of unknown moxifloxacin concentration.

#### Determination of moxifloxacin in the pharmaceutical dosage forms

Ten tablets of Avelox 400 mg were accurately weighed, crushed, mixed in a mortar. An appropriate amount (400 mg of moxifloxacin powder, from each) was weighed, transferred to a 100 ml beaker and dissolved in double distilled water, sonication for about 15 min and completed to the mark with the water. A 5.0 ml aliquots of these solutions were transferred to 50 ml standard flask, the pH was adjusted to 7.0 using tris buffer and completed to the mark with water. The potential of the solution was measured using MOX-sensors in conjunction with an Orion Ag/AgCl double junction reference electrode. The potential of the stirred solution was recorded after the signal stabilization (±0.5 mV/min) and the concentration was calculated from the previous calibration graph under identical experimental conditions from standard solutions of MOX.

Alternatively, the potentials displayed by moxifloxacin test solution before and after the addition of a 1.0 ml aliquot of 1 × 10^−3^ M moxifloxacin were measured. The change in the potential readings was recorded and used to calculate the unknown moxifloxacin concentration in the test solution using the standard addition technique [24].

Reconstituted powder: one mixture was prepared with a known amount of moxifloxacin powdered (20 mg) and other components such as starch, lactose and magnesium stearate. The accuracy of the potentiometric determination of MOX in this powdered was checked by evaluation the recovery.

## Results and discussion

Sodium tetraphenyl borate, phosphomolybdic acid, and phosphotungstic acid were tested as ion-pairing agent for the preparation of electroactive ion association complexes for MOX. Sparingly soluble complexes of MOX-TPB, MOX- PM or MOX - PT have been instantaneously formed upon the addition of MOX solution to solutions of Na TPB, PMA or PTA respectively. The dry powder of the formed ion pairs are used for the construction of a new moxifloxacin ion selective electrodes. The elemental analysis showed that the composition of the complex is 1:1 in case of MOX:TPB, 3:1 for MOX: PM or MOX-PT respectively. Plasticized polymeric membranes were prepared by using membrane cocktails with compositions 1.82% of the corresponding ionic pair (MOX-TPB or MOX-PM or MOX-PTA), 34.54% of PVC and 63.64% of the corresponding plasticizer (DBS, DOP and NPOE).

### Effect of plasticizer type on the characteristic performance of the sensors

Moxifloxacin ion-selective membrane sensors with different electroactive materials were investigated in order to compare their performance. Three reagents were investigated as possible counter ion for the preparation of the electroactive complex of MOX, namely TPB or PM or PTA were tested as ion-pair reagents. The obtained ion-pairs combined with three plasticizer, DOP, DBS and NPOE to give different combinations were tested. It is well known that the construction of PVC based ISEs required the use of a plasticizer which acts as a fluidizer allowing homogenous dissolution and diffusion mobility of the ion-pair inside the membrane. PVC membrane sensor of MOX-TPB, MOX-PM or MOX-PT with different plasticizer namely (DBS or DOP or NPOE) was found to be all suitable and optimum available mediators for MOX membrane sensors. In fact, o-NPOE was found to be the optimum available mediator for MOX-TPB, MOX-PM or MOX-PT membrane sensors (ion-associates). The use of non polar mediators such as DBS, DOP gave less solubility of the ion-pair and less response of the analyte compared with o-NPOE.(slope about 50.0 mV per concentration decade for both DBS and DOP respectively). It seems that o-NPOE improves the membrane selectivity due to its high dialectical constant (ε = 24), affects considerable dissolution of ion-association within the membrane; consequently enhances its partition coefficient in the membrane and also provided suitable mechanical property of the membrane compared with less permittivity plasticizers of DBS (ε = 4) or DOP (ε = 7) and the solubility of electroactive materials are relatively small compared with NPOE. Ortho-nitrophenyl octylether was used in case of MOX-TPB, MOX-PM or MOX-PT for carrying out other experiments in this investigation.

### Effect of pH and the response time

**Figure 2:**
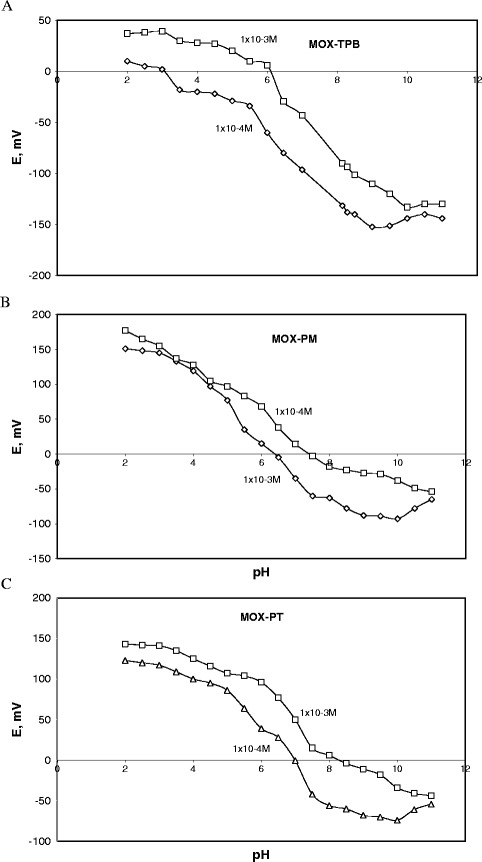
**Effect of pH on the response of moxifloxacin sensors A) MOX-TPB, B) MOX-PM and C) MOX-PT using two series of moxifloxacin solutions: 1 × 10** ^**−3**^ **M, and 1 × 10** ^**−4**^ **M.**

The electrode response for different moxifloxacin concentrations was tested at different pH values, the pH being adjusted using hydrochloric acid or sodium hydroxide. The MOX-PVC electrode dipped into MOX solution of 1 × 10^−3^ and 1 × 10^−4^ M the potential of the electrode was plotted against the pH of solution. The potentials show that the slope per concentration decade is constant (~53.0 ± 0.5 or 54.5 ± 0.5 or 55.0 ± 0.5 mV for MOX -PM or MOX -PT respectively) in the pH range of 6–9.0 (Figure 2). At higher pH values (>9.5), the potential decreased due to the gradual increase in the concentration of the unprotonated MOX.

Average response time is defined [25] as the time required for the electrode to reach a stable potential within ±1 mV of the final equilibrium value. After successive immersion of the electrode in different moxifloxacin solutions each having a 10-fold difference in concentration or after rapid 10-fold increase in concentration by addition of MOX. The response time was found to be 20s for concentration of ≥1 × 10^−3^ M and ≤ 30 s for concentration 1 × 10^−4^ M. Day-to-day reproducibility of the sensor is about ± 0.5 mV for the same solution and the useful lifetime of the sensor is 4 weeks, during which the potential slope is reproducible to within ±1 mV/ decade. Also after more than one month a new section from the master membrane was found to work with a good reproducibility.

### Effect of diverse ions

The influences of different organic and inorganic ions on the response of MOX sensors were investigated. The selectivity coefficients ${K}_{MF,B}^{Pot}$ were evaluated according to IUPAC guidelines using the separate solution method (SSM) or mixed solution method [25,26] in tris-buffer solution of pH 7.0. The selectivity coefficient ${K}_{MOX,B}^{pot}$ measured by separate solution method was calculated from the following equation:

1 $$ log{K}_{A,B}^{pot} = {E}_B-{E}_A/S + \left[\ 1 - {Z}_A/{Z}_B\right] log\ {a}_A $$

where E_A_ and E_B_ are the potential reading observed after 1 min of exposing the sensor to the same concentration of MOX and interfering species (1 × 10^−3^ each) alternatively. The symbol a_A ,_ and a_B_ are the activity of MOX and interfering species and Z_A_ and Z_B_ are the charge of moxifloxacin and interfering species and *S* is slope of calibration graph (mV/ concentration). The selectivity coefficient by mixed solution method was defined as the activity ratio of primary and interfering ions that give the same potential change under identical conditions as given in equation 3.

2 $$ {K}_{A,B}^{pot} = \left(a{`}_A - {a}_A\right)/{a}_B $$

Where a`_A_ known activity of primary ion solution added into a reference solution that contains a fixed activity (a_A_) of primary ions, and the corresponding potential change (ΔE) is recorded. Next, a solution of an interfering ion (a_B_) is added to the reference solution until the same potential change (ΔE) is recorded. The change in potential produced at the constant back ground of the primary ion must be the same in both cases. The results are given in Table 1. The results reveal reasonable selectivity for MOX in presence of many related substances.

**Table 1:** **Potentiometric selectivity coefficients of some interfering ions, using MOX**

**Interferent, J**	${\boldsymbol{K}}_{\boldsymbol{MF},\mathbf{B}}^{\boldsymbol{Pot}}$ **MOX-TPB**	${\boldsymbol{K}}_{\boldsymbol{MF},\boldsymbol{B}}^{\boldsymbol{Pot}}$ **MOX-PM**	${\boldsymbol{K}}_{\boldsymbol{MF},\boldsymbol{B}}^{\boldsymbol{Pot}}$ **MOX-PT**
**Na** ^**+a**^	1.3 × 10^−3^	1.3 × 10^−3^	1.65 × 10^−3^
**K** ^**+a**^	2.9 × 10^−3^	3.33 × 10^−3^	1.2 × 10^−4^
**Ca** ^**2+a**^	1.5 × 10^−3^	1.49 × 10^−3^	1.79 × 10^−3^
**Cu** ^**2+a**^	1.84 × 10^−3^	1.845 × 10^−3^	2.4 × 10^−2^
**Co** ^**2+ a**^	9. 5 × 10^−3^	9. 5 × 10^−3^	0.0113
**Mg** ^**2+ a**^	9.1 × 10^−3^	1.55 × 10^−3^	1.65 × 10^−2^
**Magnesium stearate** ^**b**^	2.0 × 10^−3^	2.18× 10^−3^	2.4 × 10^−3^
**Glucose** ^**b**^	2.0 × 10^−3^	2.09× 10^−3^	2.4 × 10^−3^
**Lactose monohydrate** ^**b**^	2.0 × 10^−3^	2.27× 10^−3^	2.51 × 10^−3^
**Starch** ^**b**^	2.0 × 10^−3^	2.27× 10^−3^	1.79 × 10^−3^
**Microcrystalline cellulose** ^**b**^	2.0 × 10^−3^	2.27× 10^−3^	1.79 × 10^−3^

^a^separate solution method and ^b^mixed solution method.

### Sensors characteristics

The potentiometric response characteristics of the moxifloxacin sensors based on the use of MOX-TPB, MOX-PM or MOX-PT ion pair complexes and DBS or DOP or NPOE as a plasticizer in a PVC matrixes were evaluated according to IUPAC recommendations [25]. Results in Table 2 show the characteristics performance of the PVC membrane sensors. The least squares equations obtained from the calibration data as follows:

**Table 2:** **Response characteristics of moxifloxacin-PVC matrix membrane sensors**

**Parameter**	**MOX-TPB**	**MOX-PM**	**MOX-PT**
**Slope, (mV/ decade)**	53.0 ± 0.5	54.5 ± 0.5	55.0 ± 0.5
**Intercept, mV**	137.8 ± 0.5	143.03 ± 0.5	141.78 ± 0.5
**Correlation coefficient, (r)**	0.996	0.995	0.995
**Lower limit of detection (LOD), M**	4.0 × 10^−6^	5 × 10^−6^	5 × 10^−6^
**Lower limit of quantification (LOQ), M**	3.0 × 10^−6^	4.0× 10^−6^	4.0 × 10^−6^
**Response time for 1 × 10** ^**−3**^ **M solution, s**	30 ± 0.5	30 ± 0.5	30 ± 0.5
**Working pH range**	6.0 - 9.0	6.0 - 9.0	6.0 - 9.0

3 $$ E(mV) = Slog\ \left[MOX\right] + Intercept $$

where *E*, is the potential of the electrode, *S* equal slope of the electrodes (53.0 ± 0.5, 54.5 ± 0.5 and 55.5 ± 0.5 mV for MOX-TPB, MOX-PM, and MOX-PT, respectively) and intercept (137.8 ± 0.5, 143.04 ± 0.5 and 141.78 ± 0.5 for MOX-TPB, MOX -PM, and MOX -PT, respectively).

### Validity of the proposed method

#### Limit of quantification and limit of detection

**Figure 3:**
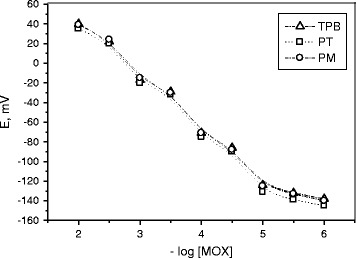
**Calibration curve of MOX membrane sensors.**

Each of different concentration of standard solution was tested five times. The potentials obtained for the five analyses were averaged at each concentration. The average potential was plotted versus concentration. The relation between potential and concentration is logarithmic (equation 1) *X* = *S* log [MOX] + *Y*, where *X* is equal the potential, *S* is the slope, and *Y* is the intercept and ( *r )* is the correlation coefficient. The sensors display a linear response over the concentration range 1 × 10^−2^ to 4 × 10^−6^, 1 × 10^−2^ to 5.0 × 10^−6^, 1 × 10^−2^ to 5.0 × 10^−6^ M), respectively over a pH range of 6.0 - 9.0. The limits of detection (LOD) and limits of quantification (LOQ) were determined according the IUPAC recommendation [24]. The lower limit of detection (LOD) defined as the concentration of MOX corresponding to the intersection of the extrapolated linear segment of the calibration graph which is of 3 × 10^−6^, 4 × 10^−6^ and 4.0 × 10^−6^ M for sensor 1, 2 and 3 respectively( Figure 3).

### Recovery

The recoveries of MOX were calculated by comparing the potential of the measured or found concentration to direct added standard in tris buffer pH 7.0. The assay of recovery, at each concentration, was computed using the following equation:

$$ Recovery\left(\%\right) = \left( found\  concentration/ added\  concentration\right) \times 100. $$

The average recovery of the direct determinations of 400 μg/ml of MOX was 98.5 , 99.1 and 98.6% with RSD value of 1.76, 1.65 and 1.78% for sensor 1 , 2 and 3 respectively (Table 3).

**Table 3:** **Day to day reproducibility of the proposed membrane sensors**

**Parameter**	Moxifloxacin (400 μg/ml)* within- day			Moxifloxacin (400 μg/ml)* within-days		
	**MOX-TPB**	**MOX-PM**	**MOX-PT**	**MOX-TPB**	**MOX-PM**	**MOX-PT**
**R,%**	98.5	99.1	98.60	98.3	99.0	98.5
**R.S.D,%**	1.76	1.65	1.78	1.9	1.7	1.9
**E,%**	1.52	0.9	1.41	1.72	1.0	1.52
**Slope**	53.0 ± 0.5	54.5 ± 0.5	55.0 ± 0.5	53.0 ± 0.6	54.5 ± 0.6	55.0 ± 0.6
**Correlation coefficient**	0.998	0.998	0.997	0.997	0.997	0.997

*Average of 5 measurements ± RSD.

*R%, recovery percentage; RSD relative standard deviation.

*E, is error% ( actual concentration –found concentration/actual concentration%).

### Precision and accuracy of the method

The intra-day, inter-day accuracy and precision of the assays were investigated [27] by the analysis of MOX at 400 μg/ml in five replicate over a period of three days. The five replicate were subject to estimate the intra-day and inter-day precision. Calibration curves were prepared and analyzed daily and linear models were used to determine concentrations in the quality control samples. Percent accuracy was determined (using the data from the precision assessment) as the closeness of found concentration to the added standards. Precision was reported as% RSD. The results obtained (Table 3) are within the acceptance range of less than 3.0% (precision) and more than 98.3% for the accuracy.

#### Ruggedness

The ruggedness of the potentiometric method was evaluated [27] by carrying out the analysis using two different analyst (operator) and different instruments on different days. The RSD of less than 3.0% were observed for repetitive measurements in three different day time periods using two different instruments and operators. The results indicate that the method is capable of producing results with high precision.

### Robustness

The robustness of the method is demonstrated [27] by the versatility of the experimental factors that affecting the potential response (e.g. pH and response time).

Preliminary inspection of the results under these various conditions suggested that the method is fairly robust, but the pH of the measuring solution should be in the range of 6.0-9.0. The optimum pH 7.0 was used using tris buffer.

### Determination of MOX in its pharmaceutical preparation

The applicability of the MOX membrane sensors for determination of the drug in the dosage forms was firstly checked by the studying the recovery of an accurate amount of pure MOX in solutions.

The analysis of 2.0 – 4000.0 μg/ml MOX solutions (in five replicate) by direct potentiometry gave an average recovery of 98.1, 98.25 and 98.51% with a relative standard deviation of 1.76, 1.65 and 1.78% for sensor 1, 2 and 3 respectively, results are shown in Table 4.

**Table 4:** **Direct determinations of moxifloxacin using PVC membrane sensors**

**Added (μg/ml)**	MOX-TPB,*				MOX-PM,*				MOX-PT,*			
	**SD**	**RSD**	**R**	**E**	**SD**	**RSD**	**R**	**E**	**SD**	**RSD**	**R**	**E**
**2.0**	0.0297	1.9	97.5	1.9	0.0197	1.9	98.0	1.8	0.0220	1.9	98.0	1.95
**4.00**	0.0297	1.9	98.1	1.8	0.0197	1.8	98.1	1.8	0.0220	1.9	98.1	1.95
**40.0**	0.1834	1.9	98.3	1.7	0.1761	1.7	99.1	1.74	0.229	1.8	98.4	1.64
**400.0**	2.9664	1.7	98.2	1.2	5.000	1.6	98.2	1.74	1.378	1.8	98.6	1.24
**1000.0**	14.1002	1.7	98.3	1.3	16.614	1.5	98.0	1.54	7.247	1.8	98.5	1.30
**4000.0**	29.234	1.5	98.3	1.4	27.229	1.4	98.1	2.05	17.117	1.5	99.5	1.40

SD: standard deviation, RSD: relative standard deviation%, R: recovery%, E: error%.

*Average of 5 measurements.

The applicability of the MOX-membrane sensors to the determination of the drug in the dosage forms was firstly checked by studying the recovery of an accurate amount of pure MOX in a reconstituted powder samples. The recovery obtained from five measurements was found to be 98.0 or 98.0 or 98.5% with a relative standard deviation of 1.7 or 1.6 or 1.7% for MOX- TPB or MOX-PM or MOX-PT respectively. On the other hand, the determination of MOX in its formulations show an average recovery of 98.0 or 98.5 or 98.5% with relative standard deviation of 1.7 or 1.6 or 1.7% for sensor 1 or 2 or 3, respectively, results are shown in Table 5.

**Table 5:** **Determination of moxifloxacin in some pharmaceutical preparations using the proposed membrane sensors**

**Preparation**	**Moxifloxacin (nominal,value)**	Proposed method* R,% (RSD,%)			**USP R,% (RSD,%)**
		**MOX-PM**	**MOX-PT**	**MOX-PT**	
**Reconstituted powder**	20 mg	98.0(1.6)	98.0 (1.5)	98.5(1.6)	98.5 (1.6)
**Avelox tablet**	400 mg	98.0 (1.7)	98.0(1.6)	98.5(1.7)	99.0 ( 1.8)

*Average of five determinations.

Results obtained for the analysis of MOX in its formulation by direct measurements using the proposed sensors and the standard USP pharmacopeia method [28] are given in Table 5. The data obtained in Table 5 proves that the potentiometeric method shows a high degree of precision and accuracy compared with US pharmacopoeia method.

### Application of MOX-PVC sensors as indictor electrodes

**Figure 4:**
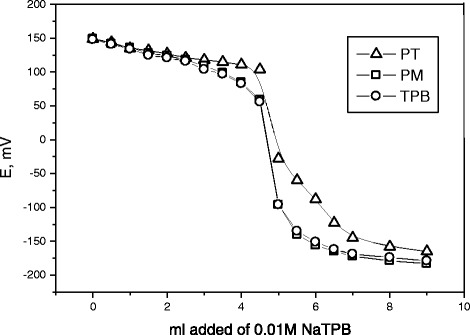
**Typical potentiometric titration curves of 5.0 ml of 1 × 10** ^**−2**^ **M MOX with 1 × 10** ^**−2**^ **M sodium tetraphenylborate using moxifloxacin membrane sensors.**

The developed electrodes in conjunction with an Ag/AgCl reference electrode have been examined as an end point indicator electrode for potentiometric titrations of the drug. Titration MOX with sodium tetraphenylborate using MOX-TPB or MOX-PM or MOX-PT sensors has been performed (Figure 4). From the results it is clear that MOX reacts with NaTPB in the molar ratio of 1:1. The titration curves were symmetrical with a very well defined potential jump of about 250 mV for MOX-TPB, MOX-PM and MOX-PT respectively, indicating the high sensitivity of the electrodes.

## Conclusion

Three different ion-pair complexes of MOX have been performed as sensors for MOX, the MOX membrane sensor displayed good analytical performance. The sensors display a fast, stable and near-Nernstian response over a relative wide moxifloxacin concentration range (1 × 10^−2^ - 4.0 × 10^−6^, 1 × 10^−2^ - 5.0 × 10^−6^, 1 × 10^−2^ - 5.0 × 10^−6^ M) for sensor 1, 2 and 3, respectively over a pH range of 6.0 - 9.0. The direct determination of moxifloxacin show an average recovery of 98.5, 99.1 and 98.6% and a mean relative standard deviation of 1.8, 1.6 and 1.8% at 400 μg/ml for sensors 1, 2 and 3 respectively. The results obtained are within the acceptance range of less than 3.0% (precision) and more than 98.3% for the accuracy. The sensors have been used as indicator electrodes for potentiometric titration of moxifloxacin.
